# DHEA down-regulates mitochondrial dynamics and promotes apoptosis of lung adenocarcinoma cells through FASTKD2

**DOI:** 10.7150/jca.93373

**Published:** 2024-02-24

**Authors:** Yan-Fei Zhang, Liu-Liu Yuan, Zong-Can Wang, Wen-Bin Zhuang, Wen-Jia Zhang, Hai-Tao Liu, Ming Li, Li-Hong Fan

**Affiliations:** 1 Nanjing Medical University, Nanjing, China.; 2 Institute of Energy Metabolism and Health, Shanghai Tenth People's Hospital, Tongji University School of Medicine, Shanghai, China.; 3 Department of Respiratory Medicine, Shanghai Tenth People's Hospital, Tongji University School of Medicine, Shanghai, China.

**Keywords:** DHEA, FASTKD2, Lung adenocarcinoma, Mitochondrial Dynamics, Reactive Oxygen Species

## Abstract

**Background:** DHEA is a steroid hormone produced by the gonads, adrenal cortex, brain, and gastrointestinal tract. While the anti-obesity, anti-atherosclerosis, anti-cancer, and memory-enhancing effects of DHEA have been substantiated through cell experiments, animal studies, and human trials, the precise mechanisms underlying these effects remain unclear. Altered mitochondrial dynamics can lead to mitochondrial dysfunction, which is closely related to many human diseases, especially cancer and aging. This study was to investigate whether DHEA inhibits lung adenocarcinoma through the mitochondrial pathway and its molecular mechanism.

**Methods:** Through animal experiments and cell experiments, the effect of DHEA on tumor inhibition was determined. The correlation between *FASTKD2* expression and DHEA was analyzed by Western blot, Reverse transcription-quantitative PCR, Immunohistochemistry, and TCGA database.

**Results:** In this study, DHEA supplementation in the diet can inhibit the tumor size of mice, and the effect of adding DHEA one week before the experiment is the best. DHEA limits the glycolysis process by inhibiting G6PDH activity, increases the accumulation of reactive oxygen species, and initiates apoptosis in the mitochondrial pathway of cancer cells.

**Conclusion:** DHEA suppresses mitochondrial fission and promotes mitochondrial fusion by downregulating the expression of FASTKD2, thereby inhibiting tumor growth and prolonging the overall survival of lung adenocarcinoma patients, which also provides a new target for the prevention and treatment of lung adenocarcinoma.

## Introduction

According to the 2023 American Cancer Statistics Report 1, the incidence and mortality of lung cancer have decreased since 1990 due to the implementation of a series of tobacco control policies and the rise of targeted therapy and immunotherapy programs. However, compared with gastric cancer, prostate cancer, pancreatic cancer and other cancers, the incidence and mortality of lung cancer are still very high. In addition, Chinese lung cancer patients also show a trend of being younger, feminine and non-smoking 2, among which lung adenocarcinoma is the main disease of non-smoking lung cancer patients.

Mitochondria are present in all eukaryotic cells and are surrounded by two layers of membrane. The outer membrane is abundant in hormone receptors, while the inner membrane governs the electron transport chain. Mitochondria, in collaboration with microorganisms, serve six crucial functions, including the regulation of ion homeostasis, biogenesis, signal transduction, energy metabolism, redox processes, and cell death. These functions collectively have a direct impact on the overall health of the body 3. Mitochondrial dysfunction is associated with many human diseases, especially cancer and aging 4.

Previous studies have shown that mitochondria are the main regulator of intracellular reactive oxygen species, which can be regulated through a variety of antioxidant pathways 5. The key pentose phosphate pathway will produce 60% NADPH to neutralize reactive oxygen species. If the mitochondria of tumor cells are dysfunctional, ROS levels will be abnormal 6. The accumulation of a large amount of ROS will directly destroy biomacromolecules and induce cellular senescence and apoptosis. Other studies have shown that mitochondrion fission and mitochondrial fusion are important features for maintaining mitochondrial and cellular functions. Dynamic changes in mitochondrial morphology and inner membrane structure can modulate the ability of cells to undergo oxidative phosphorylation and respond to energy demands 7. The imbalance of mitochondrial dynamics will inevitably lead to mitochondrial dysfunction. Many studies have shown that targeting mitochondria and cutting off the energy supply of cancer cells may be a new idea for cancer treatment 8.

DHEA is a sterol hormone derived from the mitochondria of gonadal and adrenal cortex cells, and there are corresponding receptors in mitochondria 9, 10. The plasma DHEA level began to rise in adolescence, peaked at the age of 25 years, and decreased to about 10% of the pubertal level at the age of 80 years. This trend of significant decline with age is consistent with the incidence of most cancers, and the decline in DHEA levels is thought to be associated with an increased risk of cancer 11. All studies have shown that exogenous DHEA supplements have multiple positive effects, including anti-obesity, anti-atherosclerosis, lower blood glucose levels, and enhanced memory 12. In animal experiments, DHEA can also inhibit the occurrence of chemically induced pancreatic cancer 13, breast cancer 14, lung cancer 15, liver cancer 16 and colon cancers 17, as well as the occurrence of spontaneous tumors in p53 deficient mice, and inhibit the growth of many cancer cell lines in vitro. DHEA has many protective effects, yet many of these mechanisms remain unclear.

FASTKD2 is a member of the FAST kinase domain-containing protein (FASTKD) family. Previous studies have shown that FASTKD3, another member of the FASTKD family, is essential to mitochondrial respiration, and interacts with components of mitochondrial translational apparatus and metabolic pathways 18. It has been reported that FASTKD2 interacts with such mitochondrial transcripts as 16S RNA, and MT-ND6 and MT-CO2 mRNAs, and is involved in their processing and expression. It is required for mitochondrial ribosome biogenesis. A study on hepatoma cells found that the repression of FASTKD2 expression may partly mediate the cellular effect of DHEA. Down-regulation of FASTKD2 expression in DHEA-treated cells and the ability of exogenous FASTKD2 expression to alleviate DHEA-induced growth inhibition suggest that FASTKD2 is a target for action of DHEA 19. However, the role of FASTKD2 in non-small cell lung cancer remains unclear.

Taking into consideration the pivotal role of mitochondria, this study aims to investigate the mechanism by which DHEA prevents lung adenocarcinoma occurrence, delays its progression, and improves poor prognosis. These findings may offer novel targets for intervention and drug support in the prevention and treatment of lung adenocarcinoma.

## Materials and methods

### Cell culture and reagents

Human lung adenocarcinoma cell line A549, pC9 and mouse lung cancer cell LLC were purchased from the cell bank of the Chinese Academy of Sciences. They were cultured in RPMI 1640 medium and medium supplemented with 5% fetal bovine serum respectively. They were placed in a constant temperature incubator at 37°C and 5% CO_2_. The medium was changed once every two days. All cells were used in the experiment within 10 passages or resuscitated within 1 month.

### Small interfering RNA-mediated gene silencing

*FASTKD2* siRNA was purchased from Genepharma (Shanghai, China). The target sequence for *FASTKD2* was ATGAATCACCGATCTCTTATA. Briefly, *FASTKD2* siRNA transfection was performed using Lipofectamine 3000 (Invitrogen, California, USA) according to the manufacturer's instructions. Western blot analysis and quantitative reverse transcriptase polymerase chain reaction (qRT-PCR) were used to evaluate the transfection efficiency.

### Western blotting

After drug treatment, the cells were washed with PBS for 3 times, and 100μl PMSF was added into each well of the 6-well plate. The whole cell lysate of lung adenocarcinoma cells was obtained by centrifugation at 4℃ 12000 r/min for 10min. About 20ug sample was taken for detection, and the same amount of sample was separated by sodium dodecyl sulfate-polyacrylamide gel electrophoresis and transferred to a polyvinylidene fluoride membrane. The membrane containing the target protein was incubated with the first antibody at 4℃ for more than 8 hours, and the second antibody was used to culture the membrane at room temperature for 1 hour. Western blot was detected by chemiluminescence Western blot assay kit.

### Immunohistochemistry (IHC)

The fresh tumor was fixed with 10% neutral buffered formalin overnight and embedded in paraffin. They were cut into pieces with a thickness of 5μm, dried overnight in an oven at 60°C, and stained with hematoxylin, eosin, and antibody. Each immunohistochemical staining section was scanned with panoramic MIDI and checked by K-viewer software.

### Reverse transcription-quantitative PCR (RT-qPCR)

Total RNA was isolated using the EASYspin Plus tissue/cell RNA extraction kit (Aidlab, China) according to the manufacturer's protocol. One microgram of total RNA was reverse transcribed using the HiScript Q RT SuperMix kit (Vazyme, China). Quantitative PCR was then performed using TaqMan polymerase with SYBR Green fluorescence (Nippon Gene, Japan) on a LightCycler 480 Detector (Roche, Germany). Target gene threshold cycles (Ct values) were normalized to *GAPDH* as an endogenous control. Primer sequences: *GAPDH* forward, 5'-GAAAGCCTGCCGGTGACTAA-3' and reverse, 5'-AGGAAAAGCATCACCCG GAG-3'; *Bax* forward, 5'-CCAGAGGCGGGGTTTCAT-3' and reverse, 5'-CATCCTCTGCAGCTCCATGT-3'; *Bcl2* forward, 5'-GAACTGGGGGAGGATTGTGG-3' and reverse, 5'-CATCCCAGCCTCCGTTATCC-3'); *FASTKD2* forward, 5'-TCCTGAATCCCTAAACATGAAAA-3' and reverse, 5'-GCCATAACTTCCACGAACTG-3'; *Caspase3* forward, 5'-GAAAGCCGAAACTCTTCATCAT-3' and reverse, 5'-ATGCCATATCATCGTCAGTTCC-3'.

### Flow cytometry

The lung adenocarcinoma cells were treated according to 4-5×After 24 hours of incubation with DHEA, the cells were washed twice with PBS, digested with 10% trypsin for 2-3min, and centrifuged at 800 r/min for 3min. The supernatant was removed, and then the cells were slightly resuspended. The cells were added with 2.5μl annexin V-FITC dye diluted with 50ml buffer and incubated at 37℃ for 15-20min, Then the cells were incubated with 5μl propidium iodide (PI) dye diluted with 250μl buffer for 10min at 37℃. Finally, the apoptosis rate of the control group and the experimental group was detected by flow cytometry PI and FITC channels.

### Cell proliferation test in vitro and in vivo

For cell growth assay in vitro, the same number of lung adenocarcinoma cells were seeded in 96-well plates, CCK-8 reagent was added according to the manufacturer's protocol, and the absorbance was measured at 450nm to evaluate the cell proliferation rate. In vivo tumor cell growth test, 1×10^6^ lung cancer cells LLC cells were subcutaneously injected into the right dorsal side of C57 male mice. The formula of tumor volume is: (length*width*width)/2. After 21 days, the data were weighted. The mouse protocol was approved by the Animal Protection and Utilization Committee of the Medical College of Tongji University.

### Sources of population data

Log in to the TCGA database, select TCGA-LUAD data, click files, select ht-seq TPM version of gene expression quantity data in the transcript profiling library, and then download metadata files and cart files of all lung adenocarcinoma patients. The file content includes processed clinical data and gene sequencing data. After treatment, a total of 594 samples were included, including 59 paracancerous samples and 535 cancer samples. Log on to the official website of oncomine database and input the target gene directly to query the expression of the gene in various cancers. The indicators of survival analysis included overall survival, progression-free survival and disease-specific survival.

### Statistical analysis

All basic experimental data were expressed as mean ± SD. Using graphpad prism 5 software or R language, the comparison between groups was calculated by one-way or two-way ANOVA, and *P* < 0.05 was considered to be statistically significant. Population data processing mainly uses R package such as survival and survminer in R language and MATLAB software. The main statistical methods used include t-test, rank sum test and analysis of variance, Km survival analysis, univariate Cox regression and multivariate Cox regression were used.

## Results

### Inhibitory effect of DHEA on the development of lung adenocarcinoma in vitro and in vivo

**Figure 1:**
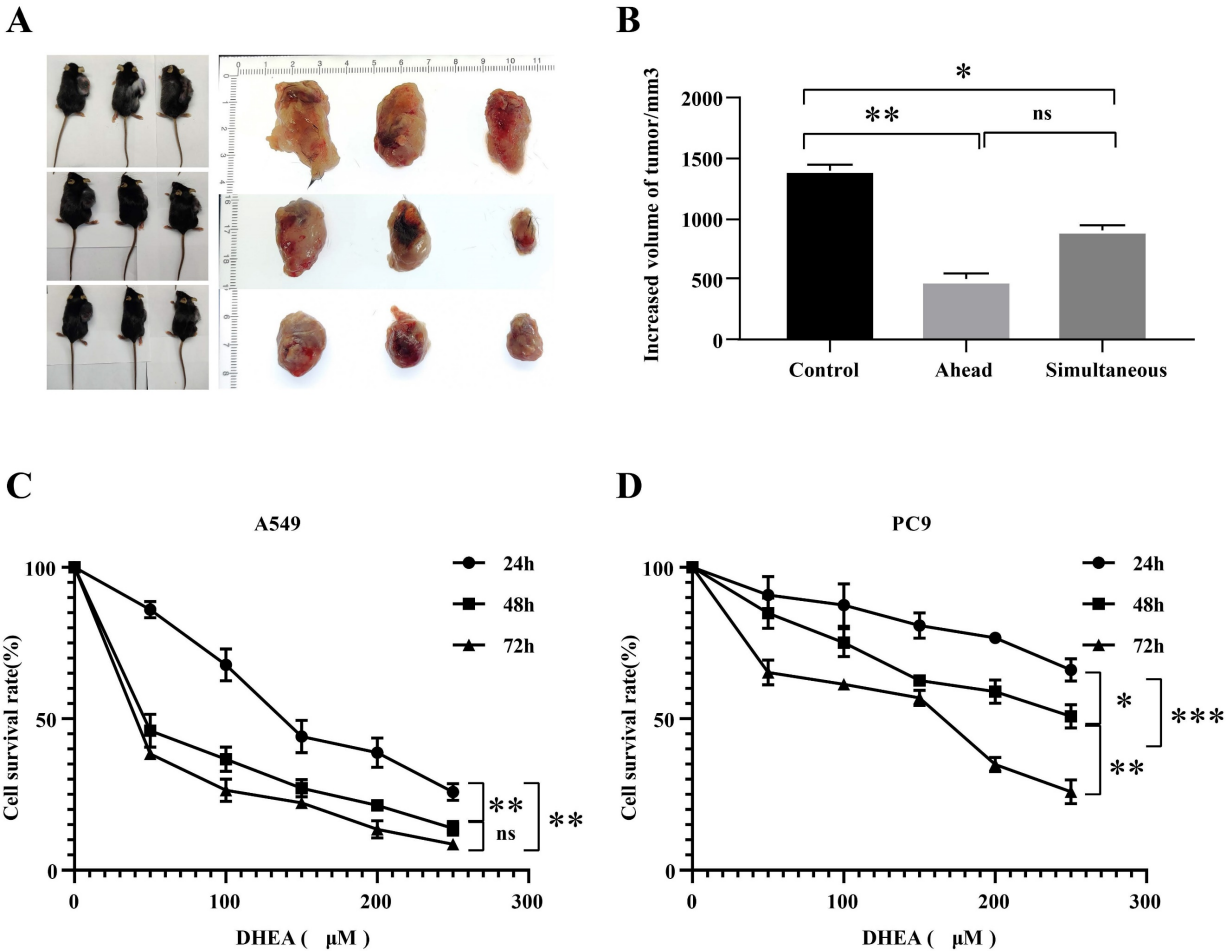
Inhibitory effect of DHEA on the development of lung adenocarcinoma in vitro and in vivo. A. Photos of mice in control group and ahead group (DHEA was supplemented one week before tumor seeding), mice in simultaneous group (DHEA was supplemented concurrently after tumor seeding) and tumor body in mice were taken. B. The tumor was recorded in the control group, the ahead group and the simultaneous group. C. The effect of DHEA on the proliferation of A549 hours. D. The effect of DHEA on the proliferation of PC9 cells within 72 hours.

In the animal experiment, as shown in Fig. 1 A-B, compared with the control group, the tumor in the ahead group could be effectively inhibited within 21 days, with the inhibition rate of 61.61% (*P* < 0.05). Compared with the control group, the tumor in the simultaneous group could be effectively inhibited within 21 days, the inhibition rate was 42.30% (*P* < 0.05). Although the effect of ahead group on tumor size was similar to that of simultaneous group. The difference was not statistically significant, but from the tumor volume, the inhibition effect of early group was better than that of simultaneous group. In cell experiment, as shown in Fig. 1 C-D, different doses of DHEA can also inhibit the proliferation of lung adenocarcinoma A549 and PC9 cells. The results showed that the tumor size in mice could be inhibited by adding a certain amount of DHEA into the diet. It is better to supplement DHEA one week before tumor seeding.

### DHEA initiated apoptosis of the mitochondrial pathway

**Figure 2:**
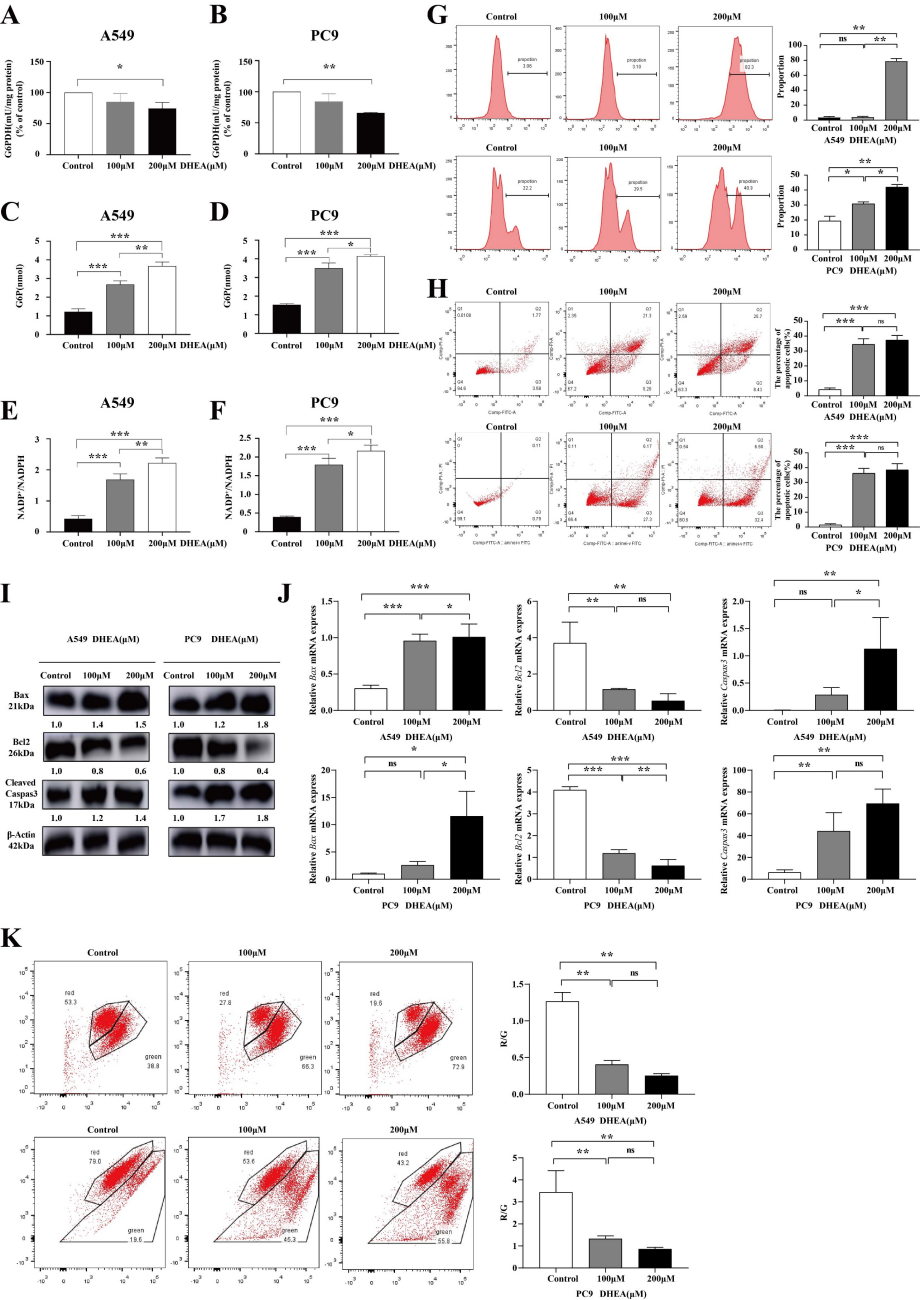
DHEA initiated the apoptosis of mitochondrial apoptotic pathway. A. The activity of G6PDH in A549 cells was changed after different concentrations of DHEA. B. The activity of G6PDH in PC9 cells was changed after different concentrations of DHEA. C. The content of G6P in A549 cells was changed after different concentrations of DHEA. D. The content of G6P in PC9 cells was changed after different concentrations of DHEA. E. The ratio of NADP+/NADPH in A549 cells was changed after different concentrations of DHEA. F. The ratio of NADP+/NADPH was changed in PC9 cells treated with different concentrations of DHEA. G. The effect of DHEA on ROS of A549 and PC9 cells within 48 hours. H. The effect of DHEA on apoptosis of A549 and pC9 cells in 48 hours. I. Western blot was used to detect the effect of different concentrations of DHEA on apoptosis related proteins within 48 hours. J. qRT-PCR was used to detect the effect of different concentrations of DHEA on apoptosis related mRNA within 48 hours. K. The effect of DHEA on mitochondrial membrane potential in A549 and PC9 cells within 48 hours.

DHEA is a noncompetitive inhibitor of G6PDH, as shown in Fig. 2 A-B, with a concentration of 100μM, the activity of G6PDH decreased by 25.63% (*P* < 0.05) and 33.91% (*P* < 0.05) in A549 and PC9 cells treated with DHEA for 48 hours. As shown in Fig. 2 C-D, compared with the intracellular G6P content (1.23 nmol) of the control group, after 100μM DHEA intervention, the content of G6P in A549 cells was as high as 2.63 nmol (*P* < 0.05), and that in PC9 cells was as high as 3.50 nmol (*P* < 0.05). The ratio of NADP+/ NADPH in A549 cells and PC9 cells was detected. NADP, as a coenzyme, participates in essential redox reactions and electron transfer in normal cellular activities 20. The NADP/NADPH ratio in a normal organism is always in a dynamic equilibrium state, which is a very important indicator that can reflect the redox state of cells, thereby determining the metabolic activity and health of a cell 21. As shown in FIG. E-F, the ratio of NADP+NADPH in A549 cells treated with 100μM DHEA was 1.63 (*P* < 0.05) and that of PC9 cells was 1.80 (*P* < 0.05). This to some extent indicates an increase in oxidative stress levels. Oxidative stress is the imbalance between the production of reactive oxygen species and the antioxidant system in vivo, which causes the damage of biological macromolecules and cell apoptosis. Therefore, we detected the level of reactive oxygen species after DHEA intervention. As shown in Fig. 2 G, after 100μM DHEA intervention, ROS in A549 cells increased by 0.35%, while that in PC9 cells increased by 11.43% (*P* < 0.05). By flow cytometry, as shown in Fig. 2 H, the apoptosis of A549 and PC9 cells could be promoted by DEHA at the concentration of 100μM and 200μM. After 48 hours of 100μM DHEA intervention, the apoptosis rate of A549 cells was 34.53% (*P* < 0.05), and that of PC9 cells was 36.29% (*P* < 0.05). As shown in Fig. 2 I-J, DHEA increased the expression of Bax and cleaved Caspas3 at protein level and mRNA level, and down-regulated the expression of BCL2. As shown in Fig. 2 K, 100μM DHEA intervention after 48 hours, the mitochondrial membrane potential of A549 cells was down-regulated by 67.86% (*P* < 0.05) and that of PC9 cells was 61.44% (*P* < 0.05). DHEA inhibited the G6PDH activity, reduced the transformation of NADP+ to NADPH, promoted the accumulation of reactive oxygen species, and started the mitochondrial dysfunction.

### DHEA down regulated the expression of FASTKD2, promoted mitochondrial fusion and inhibited mitochondrion division

**Figure 3:**
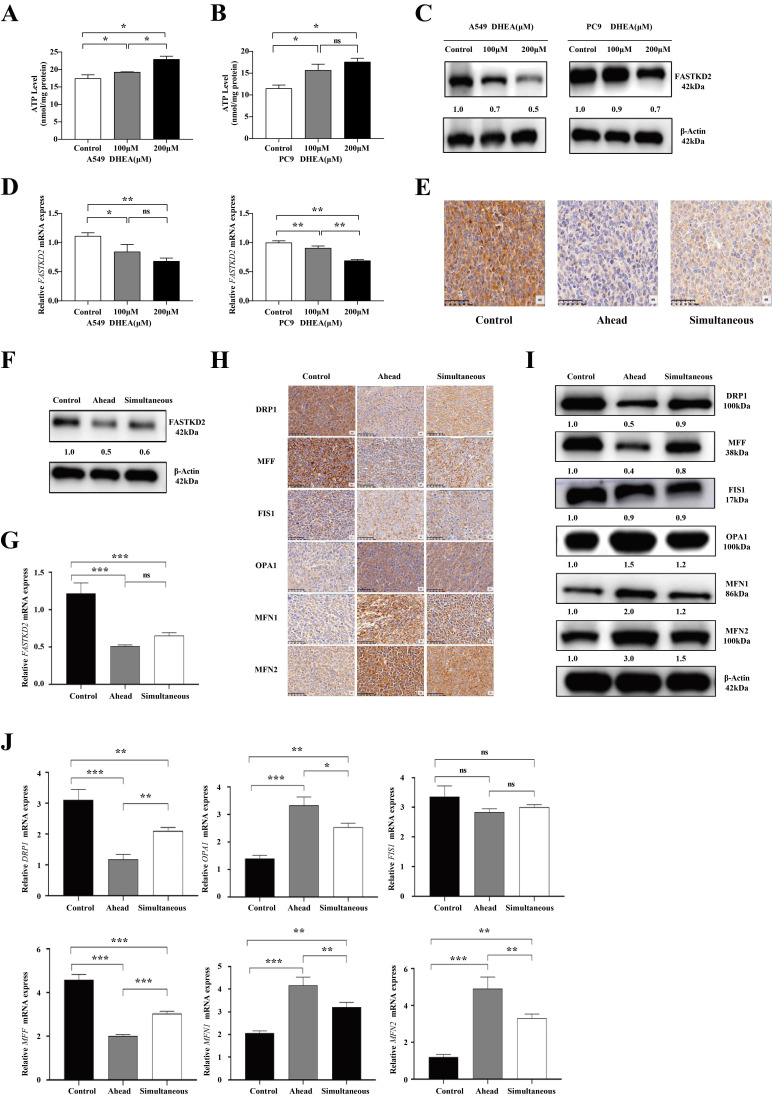
DHEA down regulated the expression of FASTKD2, promoted mitochondrial fusion and inhibited mitochondrion division. A. The changes of ATP content in A549 cells were observed after 24 hours of treatment with different concentrations of DHEA. B. The changes of ATP content in PC9 cells were observed after 24 hours of treatment with different concentrations of DHEA. C. The changes of FASTKD2 protein in A549 and PC9 cells were observed after 24 hours treatment with different concentrations of DHEA. D. After being treated with different concentrations of DHEA for 24 hours, the changes of *FASTKD2* mRNA in A549 cells and PC9 cells were observed. E. The changes of FASTKD2 protein in tumor of control group, ahead group and simultaneous group were detected. F. Western blotting was used to detect the changes of FASTKD2 protein in tumor of control group, ahead group and simultaneous group. G. qPCR was used to detect the changes of *FASTKD2* mRNA in tumor of control group, ahead group and simultaneous group. H. The changes of mitochondrial dynamics related proteins in tumor tissues of control group, ahead group and simultaneous group were studied by immunohistochemistry. I. Western blotting was used to detect the changes of mitochondrial dynamics related proteins in tumor tissues of control group, ahead group and simultaneous group. J. qPCR was used to detect the changes of mitochondrial kinetic proteins in tumor tissues of control group, ahead group and simultaneous group.

It has been reported in the literature that mitochondrial dysfunction may be associated with DHEA-induced changes in cell phenotype. Mitochondria are the main suppliers of ATP, the power center, and also the main producers of reactive oxygen species (ROS) 22, 23. As shown in Fig. 3 A-B, the ATP content of A549 cells and PC9 cells increased after 24 h of DHEA intervention. When treated with 100μM DHEA, the ATP content in A549 cells was as high as 19.27 nmol/mg protein (*P* < 0.05), and the ATP content of PC9 cells was as high as 15.69 nmol/mg protein (*P* < 0.05). The changes of mitochondrial dynamics can affect the occurrence, development and metastasis of tumors, and the expression of mitochondrial dynamics related proteins is often abnormal in tumors 24. Of mitochondrial proteins with altered expression, FAST kinase domain-containing protein 2 (FASTKD2) showed significantly reduced expression. When treated with 100μM DHEA, DHEA down regulated the expression of FASTKD2 in vitro and in vivo. In cell experiments, as shown in Fig. 3 C-D, the expression of FASTKD2 protein was consistent with that of *FASTKD2* mRNA. In the animal experiment, as shown in Fig. 3 E, the expression of FASTKD2 protein in the tumor of mice was detected by immunohistochemistry. Compared with the control group, the expression of FASTKD2 protein in the ahead group and the simultaneous group was significantly decreased. As shown in Fig. 3 F-G, Western blot and qPCR experiments were used to verify. The results showed that DHEA could down regulate the expression of FASTKD2 in tumor of mice. Although there was no significant difference between the ahead group and the simultaneous group, the data of downregulation of FASTKD2 level in the ahead group was more significant. As shown in Fig. 3 H, the expression of mitochondrial kinetics related proteins in tumor was detected by immunohistochemistry. Compared with the control group, the expression of mitochondrion related proteins DRP1 and MFF were significantly down-regulated in the ahead and simultaneous groups, but there was no significant difference in the expression of FIS1 protein. MFN1 and MFN2 fusion proteins were up-regulated in different degrees. As shown in Fig. 3I-J, Western blot and qPCR were used to verify the results. The results showed that DHEA could regulate the expression of mitochondrial kinetic proteins, inhibit mitochondrion fission and promote mitochondrial fusion. The changes of mitochondrial dynamics related proteins were more obvious in ahead group than in simultaneous group.

### DHEA promotes mitochondrial fusion and inhibits mitochondrion division by down regulating the expression of FASTKD2

**Figure 4:**
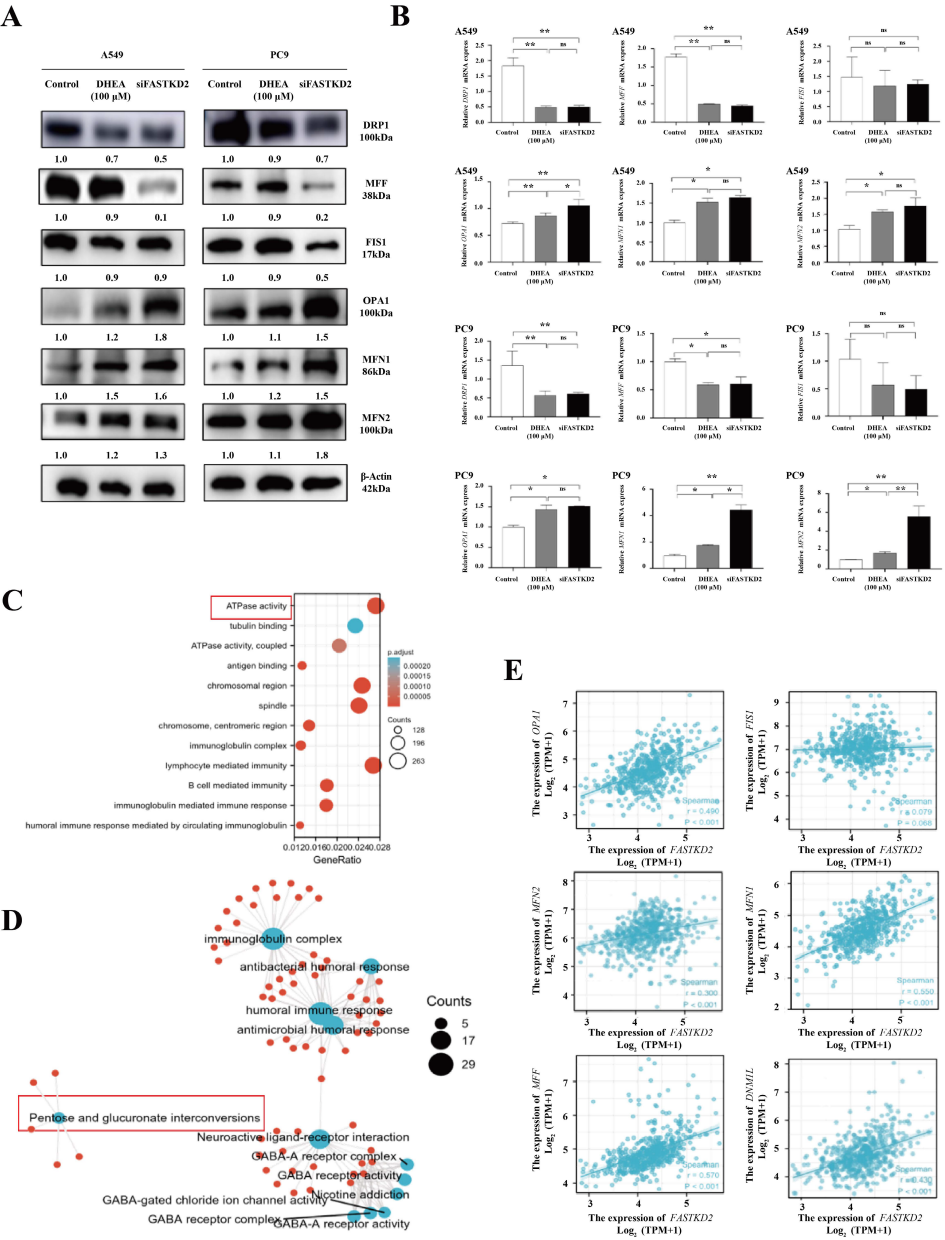
DHEA promotes mitochondrial fusion and inhibits mitochondrion division by down regulating the expression of FASTKD2. A. The expression of mitochondrial dynamics related proteins in A549 and PC9 cells was changed after 24 hours of MDEA treatment or FASTKD2 knockout. B. After 24 hours of MDEA treatment or FASTKD2 knockout, the expression of mitochondrial kinetics related mRNA in A549 cells and PC9 cells was changed. C. KEGG enrichment analysis of different genes related to *FASTKD2* in lung adenocarcinoma from TCGA database. D. Go enrichment analysis of different genes related to *FASTKD2* in lung adenocarcinoma from TCGA database. E. The correlation between the molecule *FASTKD2* and mitochondrial kinetic protein in lung adenocarcinoma data from TCGA database.

As shown in Fig. 4 A, both 100μM DHEA and FASTKD2 knockout could up regulate the expression of MFN1, MFN 2 and OPA1 at protein level, and down regulate the expression of mitochondrion related proteins DRP1 and MFF, but had no significant effect on FIS1. As shown in Fig. 4 B, this result has been further verified in qPCR experiment. In order to further prove the relationship between *FASTKD2* gene and mitochondrial dynamics related genes, the TCGA database was used to download the gene sequencing data of 59 paracancerous samples and 535 lung adenocarcinoma patients' cancer samples. All the differential genes related to *FASTKD2* were enriched and analyzed, as shown in Fig. 4 C-D, the difference genes related to *FASTKD2* regulate the activity of ATP related enzymes in human body, and are closely related to the conversion of pentose to glucuronic acid, which provides evidence for the previous basic experiments. In addition to the expression of *DRP1* and *MFF*, the expression of *MOPA1, FN1* and* MFN2* was also closely correlated with *FASTKD2*. However, it is not closely related to the expression of *FIS1*, which is basically consistent with the phenomenon found in animal experiments and cell experiments. This suggests that DHEA may inhibit cell proliferation and growth by down regulating the expression of *FASTKD2*, promoting mitochondrial fusion and inhibiting mitochondrion division.

### FASTKD2 is a predictor of lung adenocarcinoma and an independent prognostic indicator of lung adenocarcinoma specific survival

**Figure 5:**
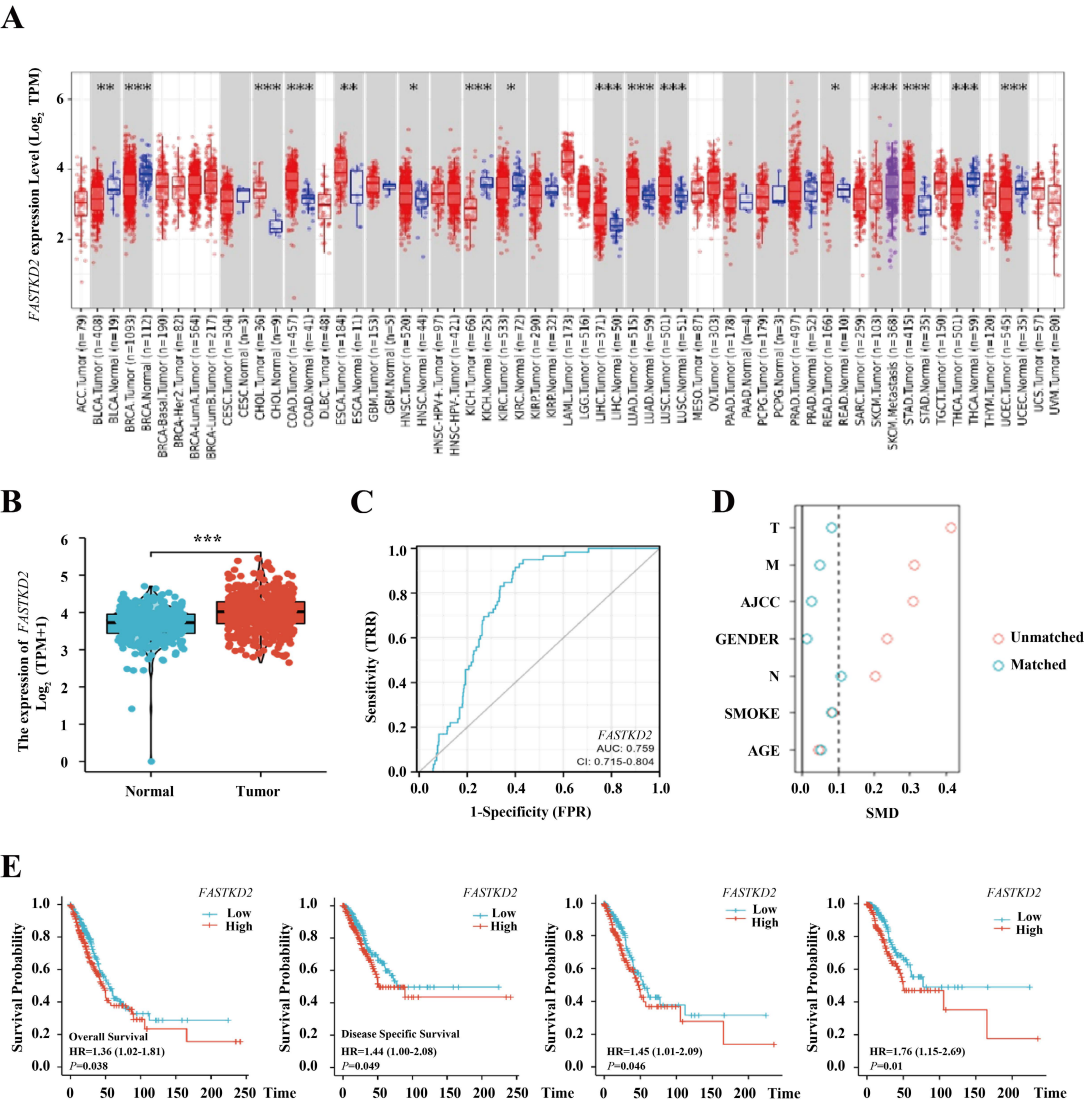
*FASTKD2* is a predictor of lung adenocarcinoma and an independent prognostic indicator of lung adenocarcinoma specific survival. A. The expression of *FASTKD2* in Pan cancer. B. *FASTKD2* was expressed in lung adenocarcinoma. C. The expression of *FASTKD2* was used as an independent ROC curve for predicting lung adenocarcinoma. D. There were differences between the two groups before and after propensity matching. E. Survival analysis before and after propensity matching.

Using the sequencing data from TCGA database, as shown in Figure 5 A, we can find that *FASTKD2* is highly expressed in a variety of cancers. As shown in Figure 5 B, this high expression is extremely significant in lung adenocarcinoma. As shown in Figure 5 C, the area under the curve reaches 0.759, the cutoff value is 4.184, the sensitivity is 0.949, and the specificity is 0.568. It is proved that the expression value of *FASTKD2* is accurate in predicting the incidence of lung adenocarcinoma. The patients were divided into high expression group and low expression group according to the median sequencing value of *FASTKD2*. As shown in Table 1, there are differences in basic information such as TNM staging among groups. Therefore, PSM propensity matching is used to balance, as shown in Figure 5 D, after matching, the difference between groups was less than 0.1, which was comparable between groups. As shown in Figure 5 E, the median survival time of patients with low expression of *FASTKD2* is higher than that of patients with high expression of *FASTKD2* whether before or after matching. Therefore, patients with high expression of *FASTKD2* have shorter overall survival time, shorter disease-specific survival time, and poor prognosis. It is confirmed that the expression value of *FASTKD2* is a predictor of prognosis of lung adenocarcinoma. As shown in Table 2 and Table 3, the univariate and multivariate Cox regression analysis further confirmed that *FASTKD2* is an independent indicator for predicting lung cancer specific survival in patients with lung adenocarcinoma. Using subgroup analysis, as shown in forest chart 5-3, *FASTKD2* has a more significant prognostic value in lung adenocarcinoma patients under 65 years of age.

## Discussion

Lung adenocarcinoma has been a serious threat to people's life and health for a long time because of its occult and rapid development. Traditional treatment methods, such as surgery, radiotherapy and chemotherapy, are not effective due to basic diseases and cancer metastasis 25. More and more evidence shows that selective delivery of drugs to specific subcellular organelles can significantly improve the efficiency of cancer treatment. The most significant one is the strategy of mitochondrial targeted therapy 26. Because mitochondria play an important role in promoting the apoptosis of cancer cells and regulating cell metabolism, this strategy is very promising in cancer treatment 27.

As early as 1981, researchers found that DHEA has anti-tumor effect, especially for breast cancer 14, prostate cancer 28 and other hormone dependent cancer 17 prevention effect is extremely significant, which has attracted extensive attention of researchers. Nevertheless, despite its potent anti-tumor efficacy against lung cancer, current research has not yielded a breakthrough. The primary objective of this study is to investigate the role of DHEA in the prevention and treatment of lung adenocarcinoma, focusing on the perspective of the six functional pathways within the mitochondria. The aim is to identify novel intervention targets within the mitochondrial framework and supportive drugs tailored for mitochondrial-focused cancer treatment. This research seeks to uncover new applications for DHEA and elucidate the specific mechanisms behind its robust functionality. Studies have shown that DHEA is a noncompetitive inhibitor of G6PDH. G6PDH is the first rate limiting enzyme in the pentose phosphate pathway. It controls the conversion of glucose-6-phosphate to glucosinolide-6-phosphate and NADP+ to NADPH 29. It plays a key role in the formation of nucleotide precursor and the maintenance of redox homeostasis. But different types of cancer, the sensitivity of this feature is different, so many experts and scholars question it. Our study confirmed that in lung adenocarcinoma A549 and PC9 cells, DHEA can inhibit the activity of G6PDH through noncompetitive inhibition, thus inhibiting the conversion of NADP+ to NADPH, reducing the production of NADPH, leading to the disorder of reactive oxygen species clearance and initiating the apoptosis process of mitochondrial pathway.

However, inhibition of pentose phosphate pathway may compensate for the loss of NADPH, which is a dynamic equilibrium network 30. Therefore, we have further explored the possible mechanism of DHEA in preventing and inhibiting cancer through mitochondrial pathway, and creatively explored a new target, FASTKD2. The relationship between *FASTKD2* gene and mitochondrial dynamics related genes was found. FASTKD2 belongs to the protein family containing fast kinase domain, which is located in mitochondria 31. As a component of functional protein ribonucleic acid module, *FASTKD2* is an essential gene for translation and transcription in mitochondria. Our study shows that DHEA can down regulate the expression of FASTKD2, it can promote the fusion of mitochondria, inhibit the mitochondrion division, disturb the balance of mitochondrial dynamics, and make the compensation of other pathways unable to continue, resulting in the accumulation of reactive oxygen species in tumor cells, and inhibit the proliferation and growth of lung adenocarcinoma cells. The present finding validates the strong correlation between DHEA and both mitochondrial function and dynamics, providing compelling evidence for the inhibitory effect of DHEA on lung adenocarcinoma development and progression through the mitochondrial pathway. In order to further explore the role of FASTKD2, we evaluated the value of high expression of FASTKD2 as a predictor of lung adenocarcinoma for the first time, with a sensitivity of 0.949, which is particularly conducive to the screening of lung adenocarcinoma. It is the first time to confirm that FASTKD2 is an independent prognostic indicator of lung adenocarcinoma. The findings suggest that FASTKD2 represents a promising therapeutic target for mitochondrial cancer, while DHEA exhibits potential as an adjunctive agent in the treatment of mitochondrial cancer.

## Conclusion

Therefore, the inhibitory effect of DHEA on lung adenocarcinoma cells is unique, it is a unique function that does not depend on human metabolism and regulation. At the same time, we have not found the safe effect of DHEA on the proliferation of bronchial epithelial cells. Therefore, at the cellular level, 100μM DHEA has no obvious harmful effect on human lung cells, and this dose of DHEA has certain safety. However, there are still some deficiencies in our study. The clinical application of DHEA still needs more randomized controlled trials to verify, and more toxicological experiments are needed to ensure its safety.

## Figures and Tables

**Table 1:** Difference of baseline indexes of lung adenocarcinoma patients before and after matching in TCGA database

Baseline indicators	Before matching				After matching		
	*FASTKD2* low expression group	*FASTKD2* high expression group	*P*		*FASTKD2* low expression group	*FASTKD2* high expression group	*P*
N	267	268			188	188	
T, n (%)			< 0.001				0.896
T1	108 (20.3%)	67 (12.6%)			57(15.2%)	60(16.0%)	
T2	129 (24.2%)	160 (30.1%)			107(28.5%)	104(27.7%)	
T3	25 (4.7%)	24 (4.5%)			20(5.3%)	18(4.8%)	
T4	4 (0.8%)	15 (2.8%)			4(1.1%)	6(1.6%)	
M, n (%)			0.035				0.902
M0	190 (47.1%)	188 (46.7%)			182(48.4%)	182(48.4%)	
M1	6 (1.5%)	19 (4.7%)			6(1.6%)	6(1.6%)	
AJCC staging, n%			0.010				0.996
Phase I	157 (29.8%)	137 (26%)			109 (29%)	107 (28.5%)	
Phase II	64 (12.1%)	59 (11.2%)			47 (12.5%)	49 (13%)	
Phase III	35 (6.6%)	49 (9.3%)			26 (6.9%)	26 (6.9%)	
Stage IV	6 (1.1%)	20 (3.8%)			6 (1.6%)	6 (1.6%)	
Gender, n%			0.027				1.000
female sex	156 (29.2%)	130 (24.3%)			88 (23.4%)	87 (23.1%)	
Male	111 (20.7%)	138 (25.8%)			100 (26.6%)	101 (26.9%)	
Age, n%			0.725				0.680
<=65	125 (24.2%)	130 (25.2%)			88(23.4%)	93(24.7%)	
>65	133 (25.8%)	128 (24.8%)			100(26.6%)	95(25.3%)	
Smoking or not			0.556				0.751
no	40 (7.7%)	35 (6.7%)			32(8.5%)	29(7.7%)	
yes	218 (41.8%)	228 (43.8%)			156(41.5%)	159(42.3%)	

**Table 2:** Univariate and multivariate analysis of overall survival predicted by *FASTKD2*.

Category	Total number of people (n)	Single factor analysis			Multivariate analysis	
		Hazard ratio (95% CI)	*P* value		Hazard ratio (95% CI)	*P* value
FASTKD2	503					
High expression	251	Reference				
Low expression	252	0.696 (0.519-0.935)	**0.016**		0.810 (0.592-1.107)	0.186
GENDER	503					
Male	235	Reference				
female sex	268	0.938 (0.700-1.256)	0.666			
AJCC staging	503					
Phase I	280	Reference				
Phase II	118	2.114 (1.466-3.049)	**<0.001**		1.003 (0.542-1.854)	0.993
Phase III	79	3.268 (2.245-4.756)	**<0.001**		1.545 (0.629-3.795)	0.343
Stage IV	26	3.461 (2.005-5.974)	**<0.001**		6.516 (0.600-70.729)	0.123
M	503					
M0	336	Reference				
M1	25	2.021 (1.181-3.457)	**0.010**		0.291 (0.027-3.115)	0.308
MX	142	0.809 (0.565-1.160)	0.250		0.906 (0.624-1.314)	0.602
N	503					
N3	14	Reference				
N0	330	0.856 (0.314-2.334)	0.761		1.394 (0.429-4.535)	0.581
N1	90	1.965 (0.709-5.449)	0.194		2.906 (0.843-10.017)	0.091
N2	69	2.555 (0.913-7.155)	0.074		2.501 (0.674-9.287)	0.171
T	503					
T1	170	Reference				
T2	266	1.553 (1.087-2.218)	**0.015**		1.260 (0.868-1.828)	0.224
T3	49	2.862 (1.713-4.784)	**<0.001**		2.289 (1.230-4.258)	**0.009**
T4	18	3.242 (1.706-6.158)	**<0.001**		1.517 (0.709-3.244)	0.283
Smoking	503					
yes	421	Reference				
no	82	1.082 (0.745-1.572)	0.679			
Age	503					
>65	256	Reference				
≤65	247	0.841 (0.628-1.128)	0.248			

**Table 3:** Univariate and multivariate analysis of* FASTKD2* in predicting lung cancer specific survival time.

Category	Total number of people (n)	Single factor analysis			Multivariate analysis	
		Hazard ratio (95% CI)	*P* value		Hazard ratio (95% CI)	*P* value
FASTKD2	503					
High expression	251	Reference				
Low expression	252	0.500 (0.350-0.714)	**<0.001**		0.562 (0.386-0.818)	**0.003**
GENDER	503					
Male	235	Reference				
female sex	268	0.910 (0.647-1.281)	0.590			
AJCC staging	503					
Phase I	280	Reference				
Phase II	118	2.217 (1.453-3.381)	**<0.001**		1.187 (0.582-2.418)	0.637
Phase III	79	3.008 (1.912-4.732)	**<0.001**		1.673 (0.573-4.884)	0.347
Stage IV	26	3.771 (2.044-6.956)	**<0.001**		8.033 (0.618-104.471)	0.111
M	503					
M0	336	Reference				
M1	25	2.250 (1.229-4.118)	**0.009**		0.260 (0.021-3.284)	0.298
MX	142	0.929 (0.617-1.400)	0.725		1.075 (0.702-1.645)	0.741
N	503					
N3	14	Reference				
N0	330	0.869 (0.273-2.767)	0.812		1.519 (0.357-6.472)	0.572
N1	90	1.939 (0.597-6.301)	0.271		2.750 (0.609-12.417)	0.188
N2	69	2.389 (0.724-7.880)	0.153		2.358 (0.484-11.481)	0.288
T	503					
T1	170	Reference				
T2	266	1.585 (1.044-2.408)	**0.031**		1.197 (0.773-1.853)	0.420
T3	18	3.108 (1.426-6.771)	**0.004**		1.359 (0.549-3.367)	0.507
T4	49	2.765 (1.521-5.026)	**<0.001**		1.997 (0.956-4.170)	0.066
Smoking	503					
yes	421	Reference				
no	82	0.922 (0.583-1.458)	0.728			
Age	503					
>65	256	Reference				
≤65	247	0.817 (0.580-1.151)	0.248			

**Table 4:**
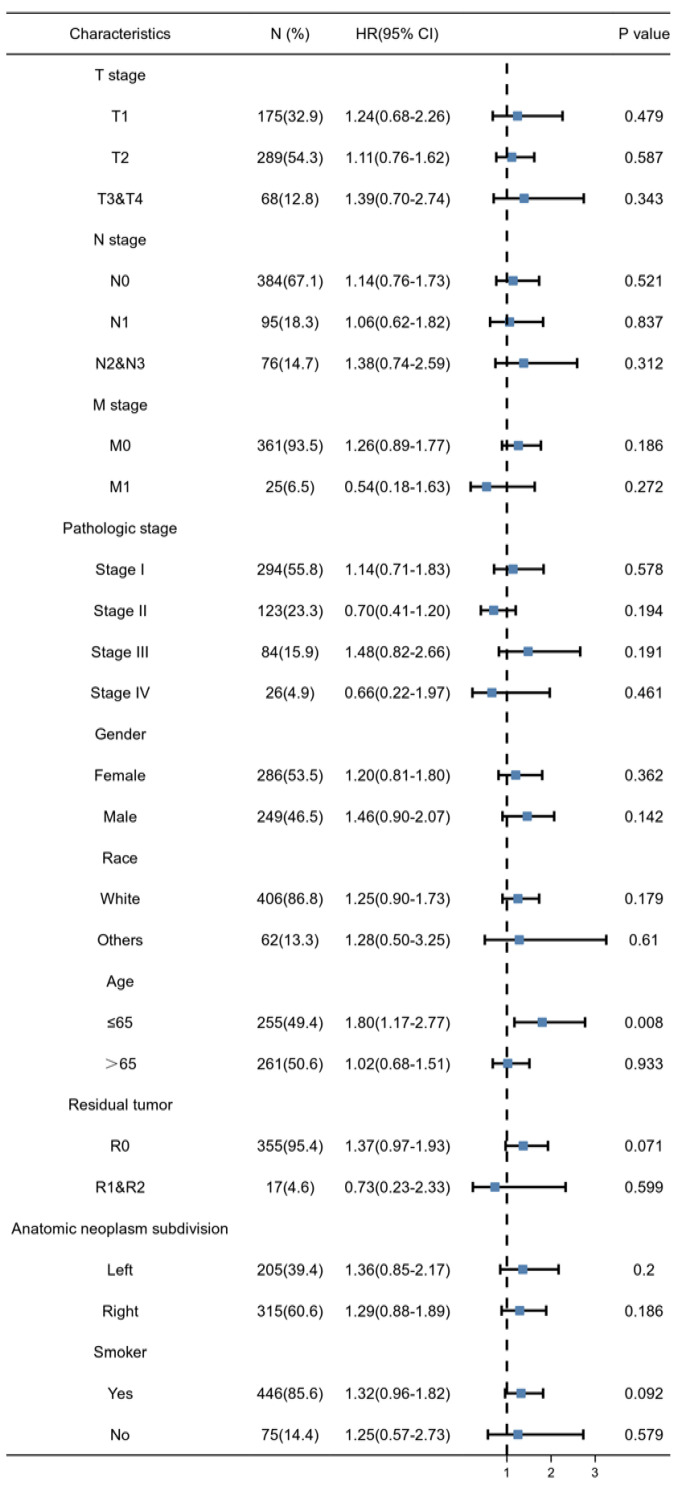
Subgroup analysis of the effect of *FASTKD2* on prognosis of patients with lung adenocarcinoma
